# Supplementary material for: Hierarchical crack-resistant, tissue-mimetic hydrogels enabled by progressive nanocrystallization of anisotropic polymer networks
Source: Nat Commun. 2025 Dec 4;16:10890. doi: 10.1038/s41467-025-65917-3 (PMC12678598; doi:10.1038/s41467-025-65917-3)
Supplement: Supplementary file 1 — Supplementary Information [file 41467_2025_65917_MOESM1_ESM.pdf]

Supplementary Information

Hierarchical Crack-Resistant, Tissue-Mimetic Hydrogels Enabled by Progressive Nanocrystallization of Anisotropic Polymer Networks

Huamin Li[1], Haidi Wu[1], Cheng Guan[1], Wenjie Hu[1], Wenwen Su[1], Dingdong Chen[1], Jiefeng Gao*[1]

[1] School of Chemistry and Materials, Yangzhou University, No 180, Road Siwangting, Yangzhou, Jiangsu, 225002, China

*Corresponding author: E-mail address: jfgao@yzu.edu.cn

Supplementary Figures

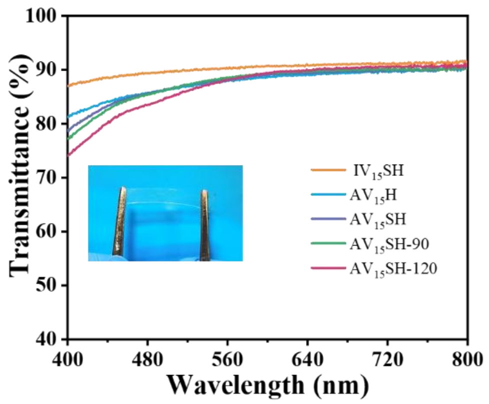

Figure S1. Photograph and UV-vis transmittance spectra showing the transparency of the hydrogels.

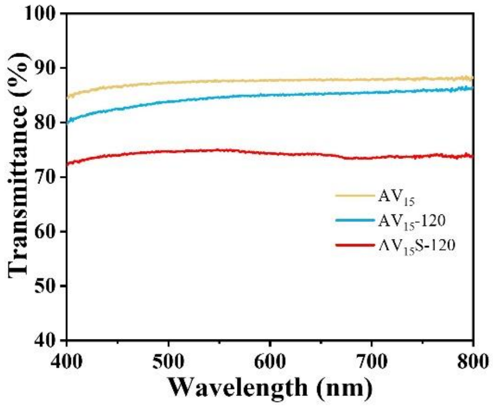

Figure S2. UV-vis transmittance spectra showing the transparency of AV_{15}, AV_{15}-120 and AV_{15}S-120.

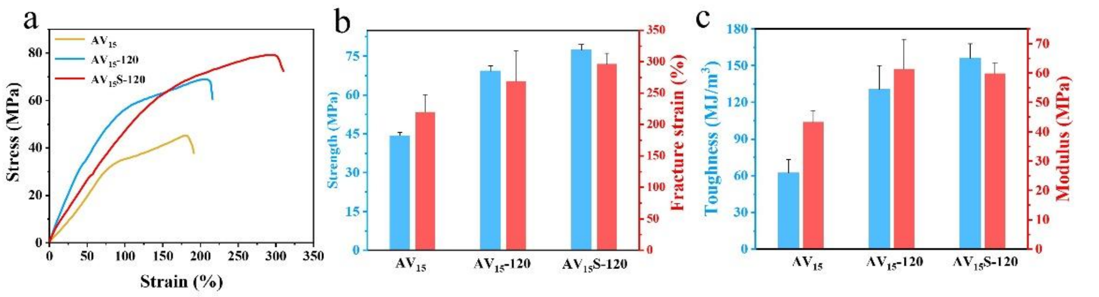

Figure S3. a) Tensile stress-strain curves of AV_{15}, AV_{15}-120 and AV_{15}S-120, with a summary of their b) tensile strength and fracture strain, and c) modulus and toughness. All data are presented as mean values ± SD, n = 3 independent samples.

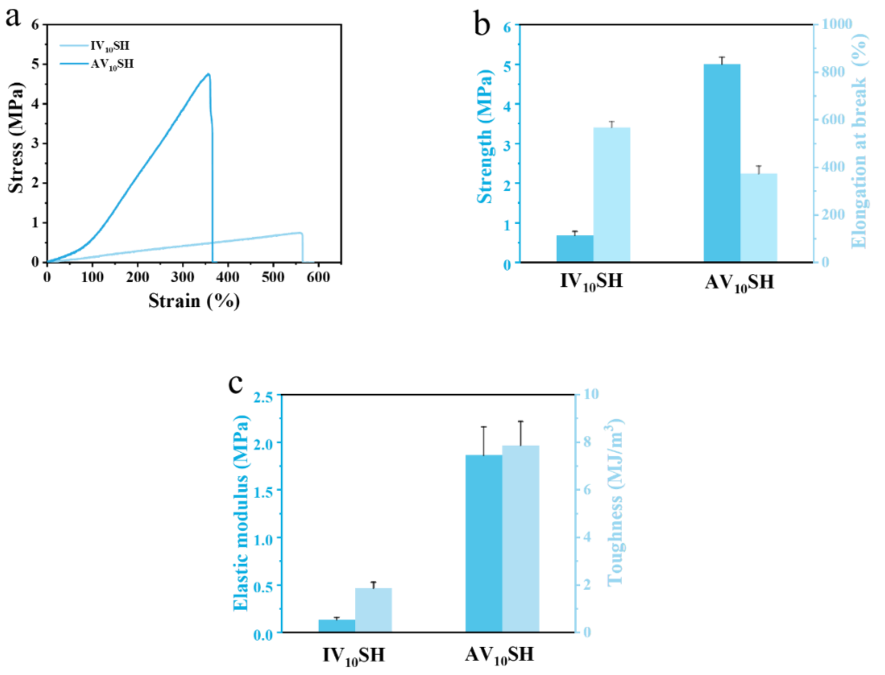

Figure S4. a) Stress-strain curves of IV_{10}SH and AV_{10}SH prepared with PVA concentration of 10 wt.%. b) Tensile strength and elongation at break (10 wt.%). c) Elastic modulus and toughness (10 wt.%). All data are presented as mean values ± SD, n = 3 independent samples.

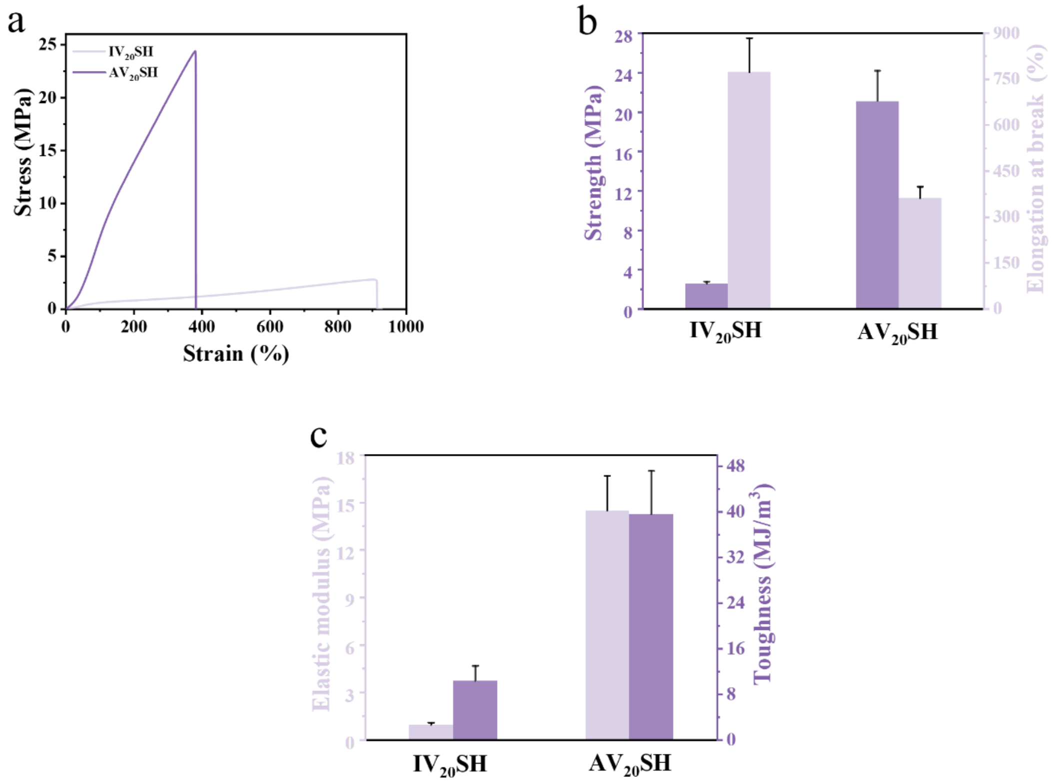

Figure S5. a) Stress-strain curves of $IV_{20}SH$ and $AV_{20}SH$ prepared with PVA concentration of 20 wt.%. b) Tensile strength and elongation at break (20 wt.%). c) Elastic modulus and toughness (20 wt.%). All data are presented as mean values ± SD, n = 3 independent samples.

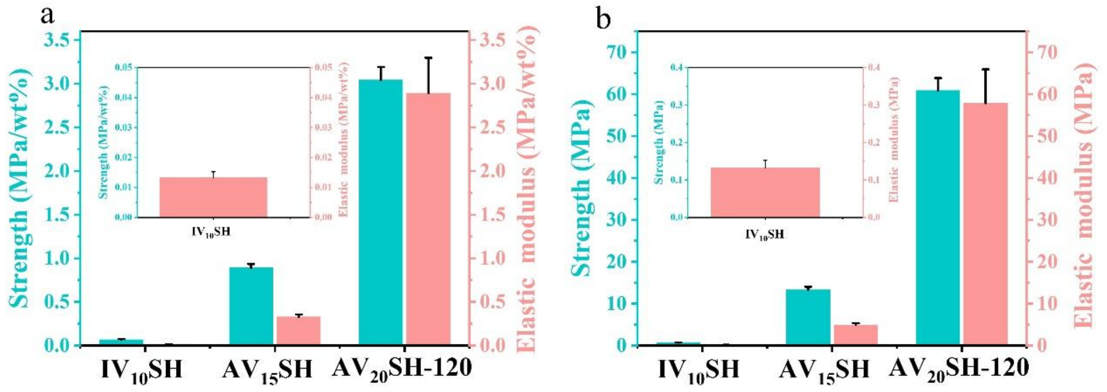

Figure S6. a) Summary of strength and modulus of different hydrogels. b) Summary of strength and modulus of different hydrogels normalized by polymer content. All data are presented as mean values ± SD, n = 3 independent samples.

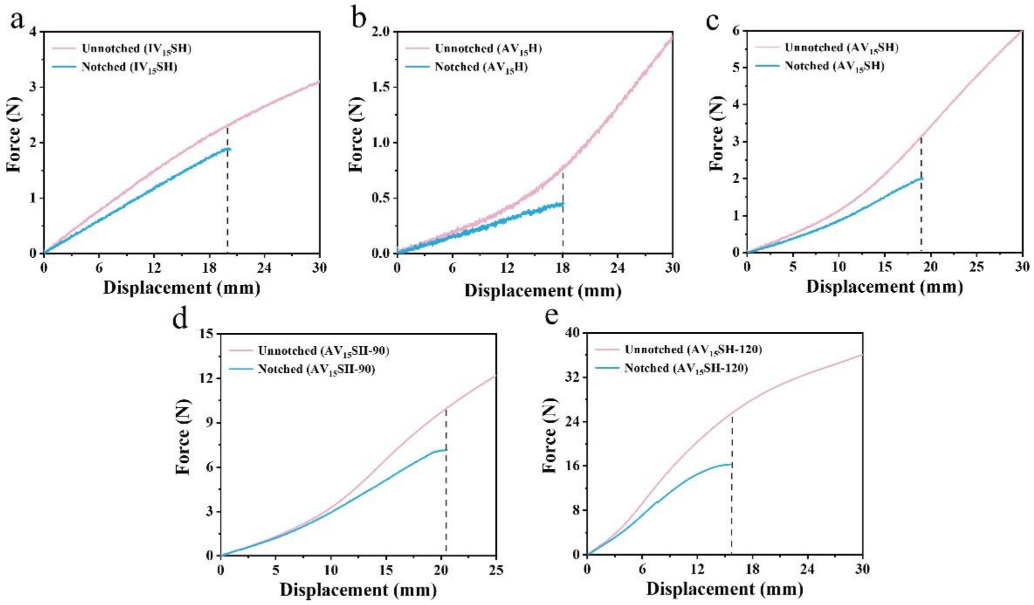

Figure S7. The force-displacement curves of unnotched and notched PVA hydrogels. a) IA$_{15}$SH, b) AV$_{15}$H, c) AV$_{15}$SH, d) AV$_{15}$SH-90 and e) AV$_{15}$SH-120.

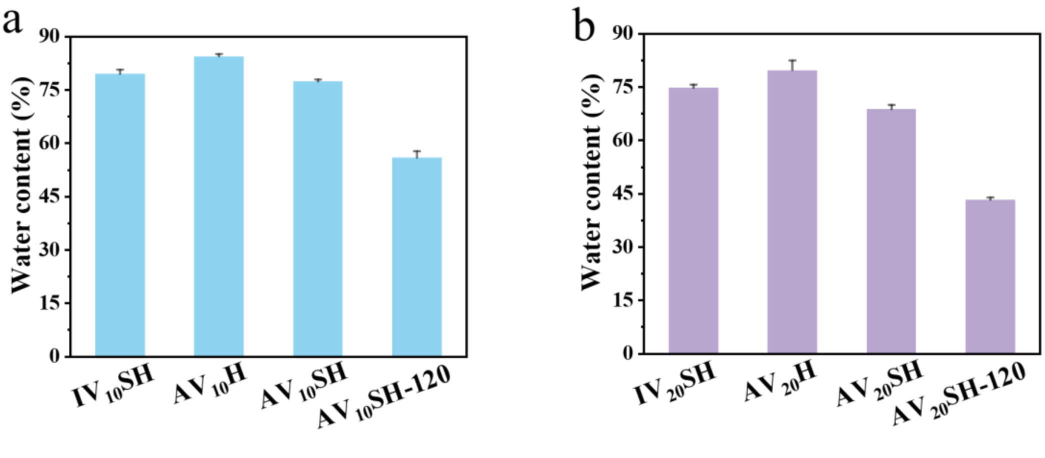

Figure S8. a) Water content of different hydrogels prepared with PVA concentration of a) 10 wt.% and b) 20 wt.%. All data are presented as mean values ± SD, n = 3 independent samples.

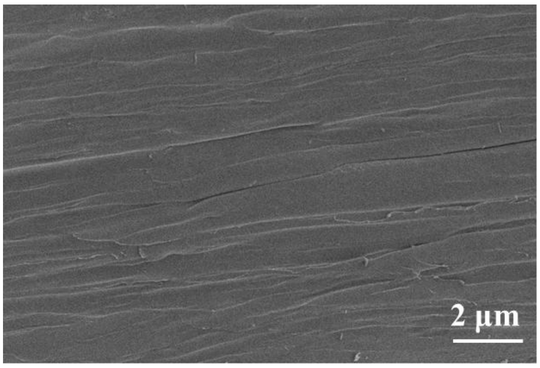

Figure S9. SEM image of $AV_{15}H$-120.

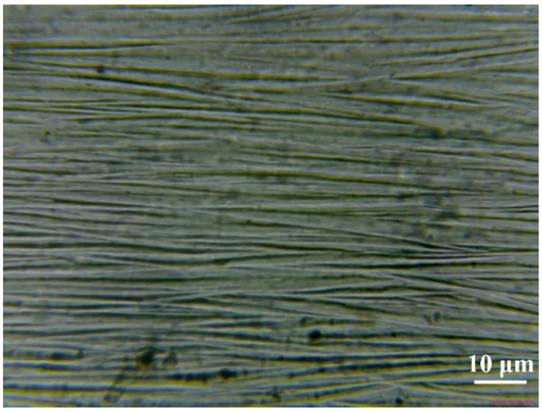

Figure S10. Optical microscope image of $AV_{15}SH$-120.

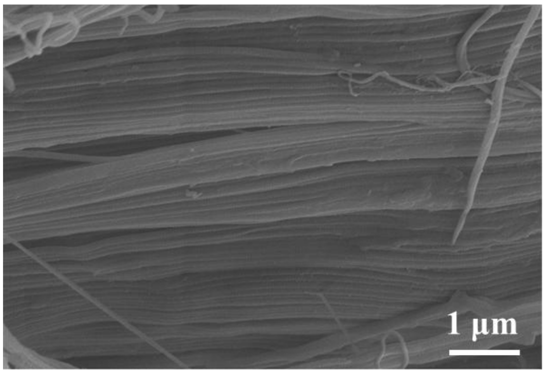

Figure S11. SEM image of tendon.

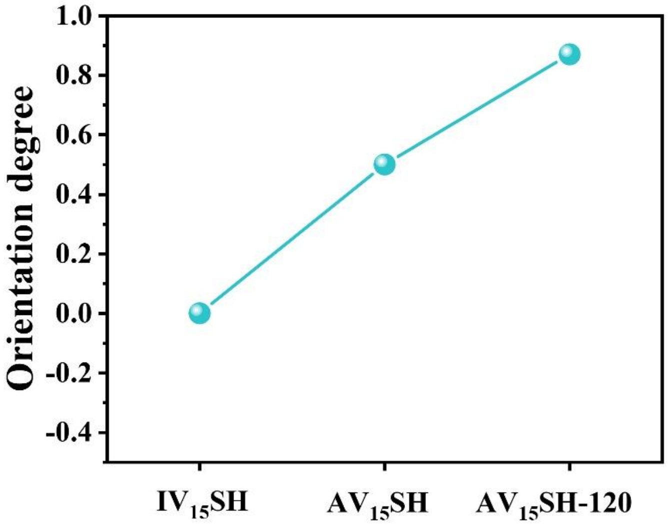

Figure S12. The orientation degree of $IV_{15}SH$, $AV_{15}SH$ and $AV_{15}SH$-120 calculated from 2D WAXS patterns.

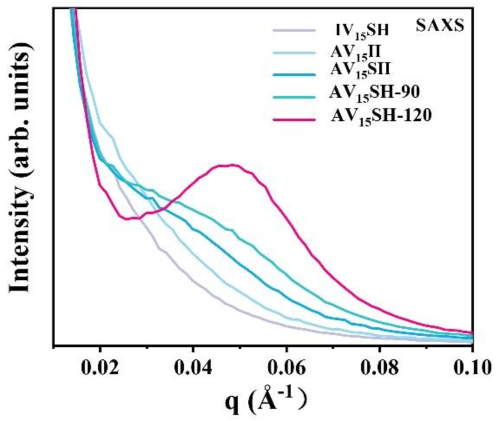

Figure S13. 1D SAXS curves (Parallel to the orientation direction, V//), depicting scattering intensity vs. scattering vector (q).

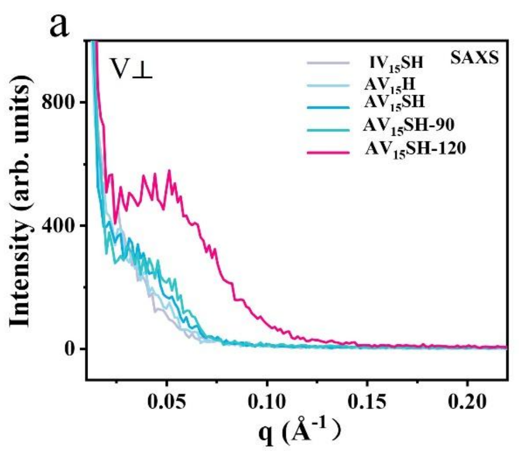

Figure S14. a) 1D SAXS curves (Perpendicular to the orientation direction, V⊥), depicting scattering intensity vs. scattering vector (q). b) 1D SAXS curves (V⊥), depicting scattering intensity vs. scattering vector (Iq²).

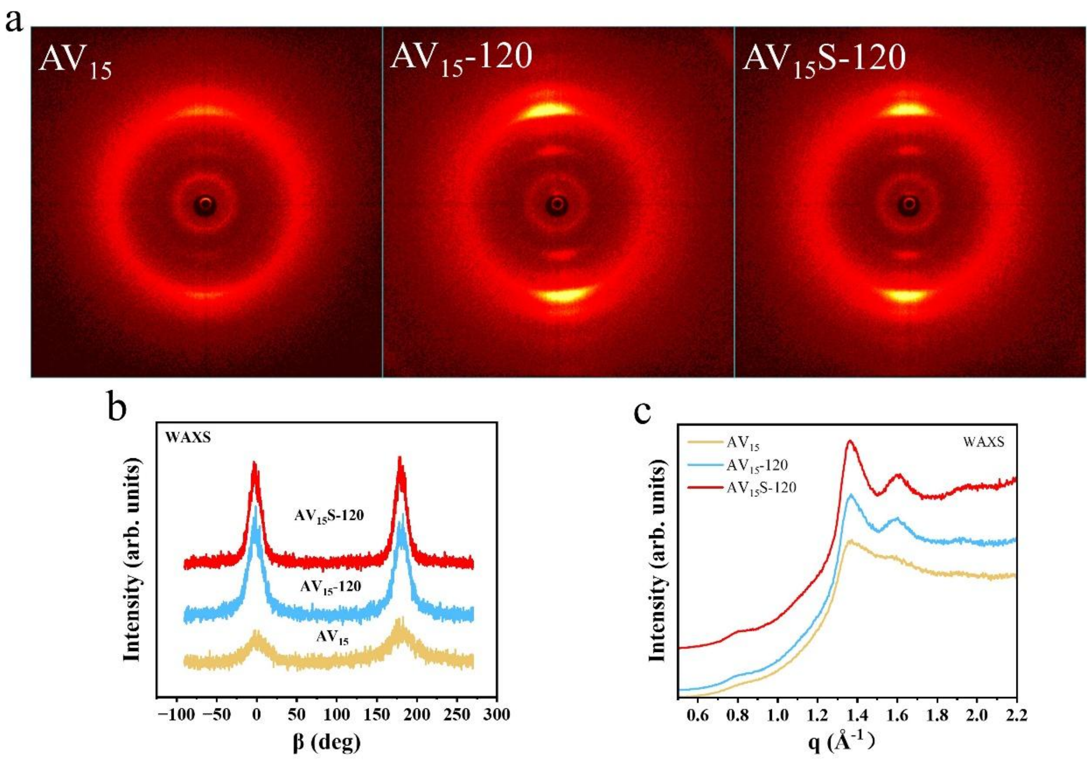

Figure S15. a) Wide-angle X-ray scattering (WAXS) patterns of AV_{15}, AV_{15}-120 and AV_{15}S-120. b) Azimuthally integrated intensity distribution of 2D WAXS patterns. c) 1D WAXS curves showing scattering intensity vs. scattering vector (q).

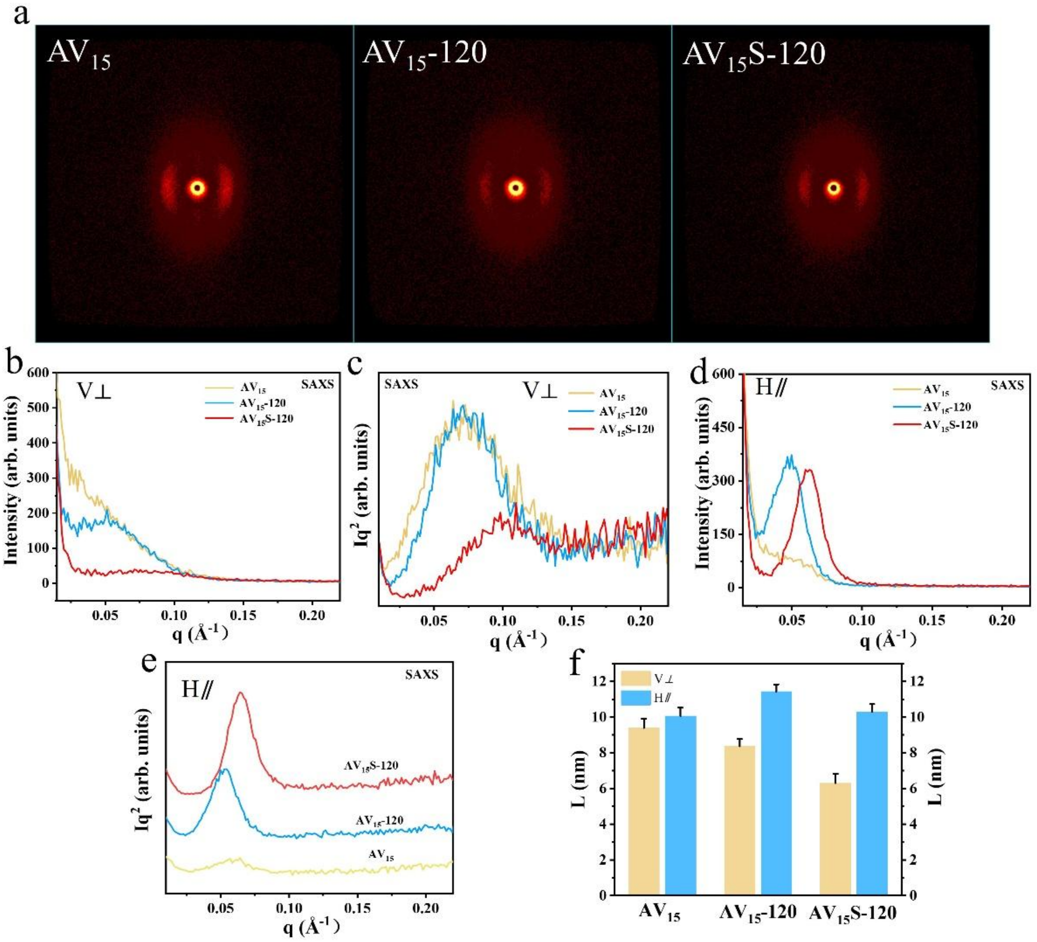

Figure S16. a) Small-angle X-ray scattering (SAXS). b) 1D SAXS curves (Perpendicular to the orientation direction, V⊥), depicting scattering intensity vs. scattering vector (q). c) 1D SAXS curves (V⊥), depicting scattering intensity vs. scattering vector (*Iq²*). d) 1D SAXS curves (Parallel to the orientation direction, V//), depicting scattering intensity vs. scattering vector (q). e) 1D SAXS curves (V//), depicting scattering intensity vs. scattering vector (*Iq²*). f) Calculated average distance between crystalline regions. All data are presented as mean values ± SD, n = 3 independent samples.

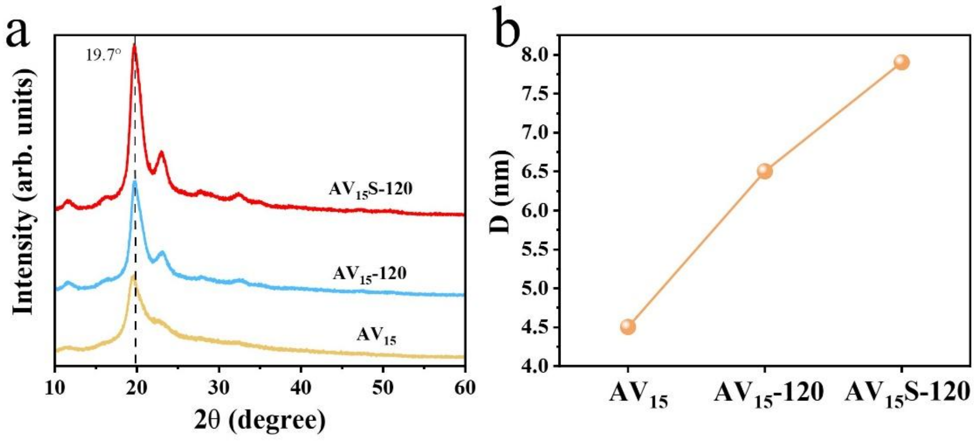

Figure S17. a) X-ray diffraction (XRD) patterns of AV_{15}, AV_{15}-120 and AV_{15}S-120. b) Summary of average size of crystalline domains of AV_{15}, AV_{15}-120 and AV_{15}S-120.

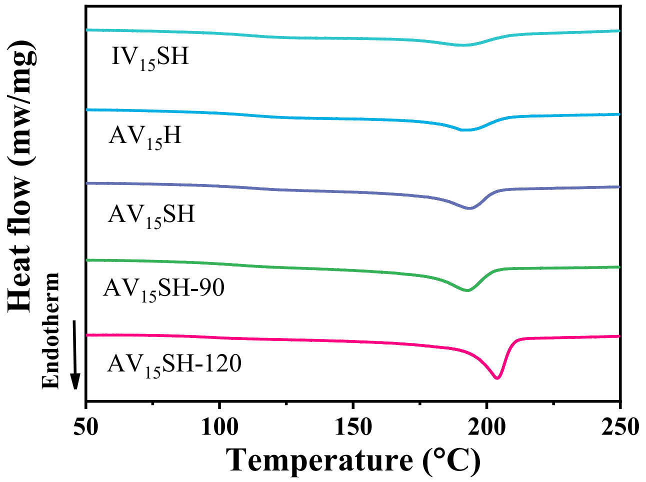

Figure S18. Differential scanning calorimetry (DSC) curves of hydrogels.

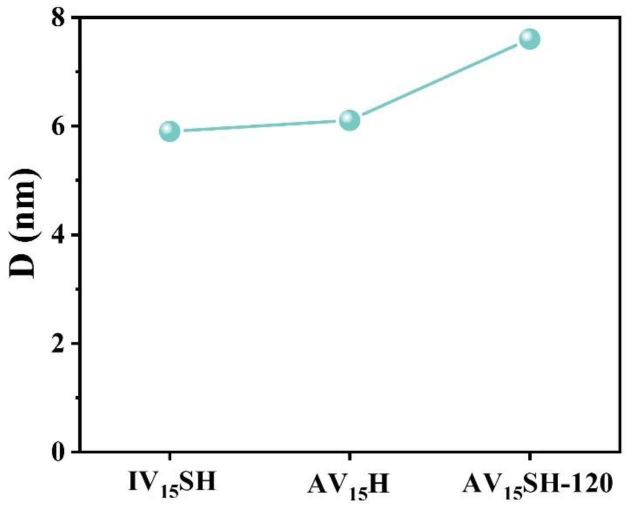

Figure S19. Summary of average size of crystalline domains of $IV_{15}SH$, $AV_{15}SH$ and $AV_{15}SH$-120.

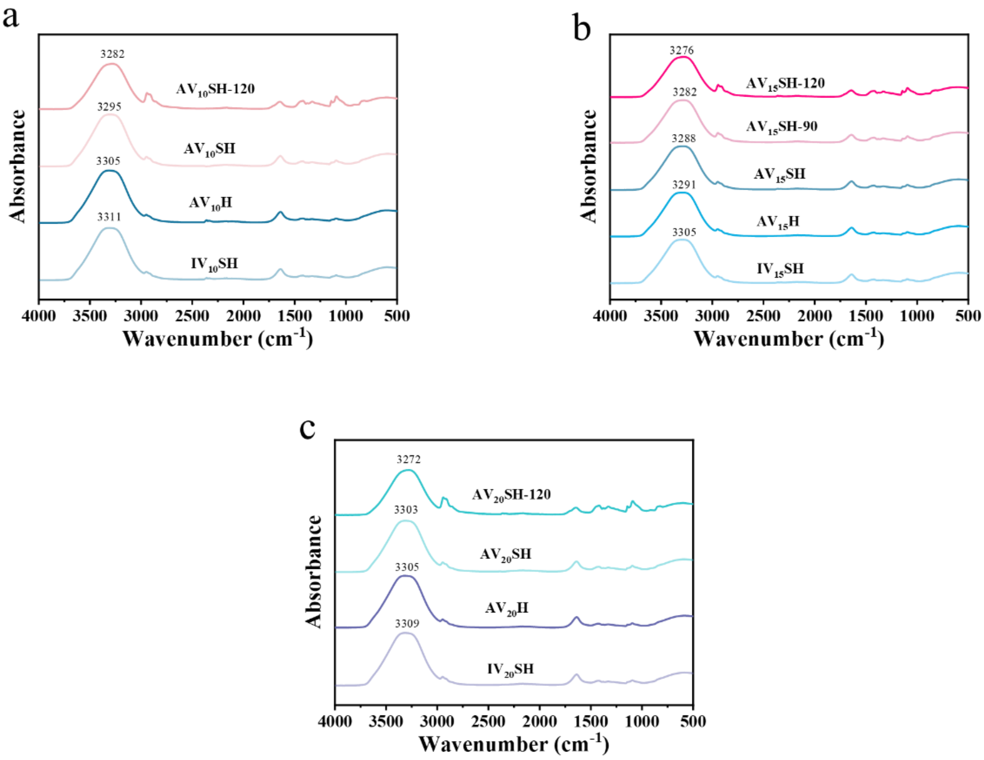

Figure S20. ATR-FTIR spectra of different hydrogels prepared with PVA concentration of a) 10 wt.%, b) 15 wt.% and c) 20%.

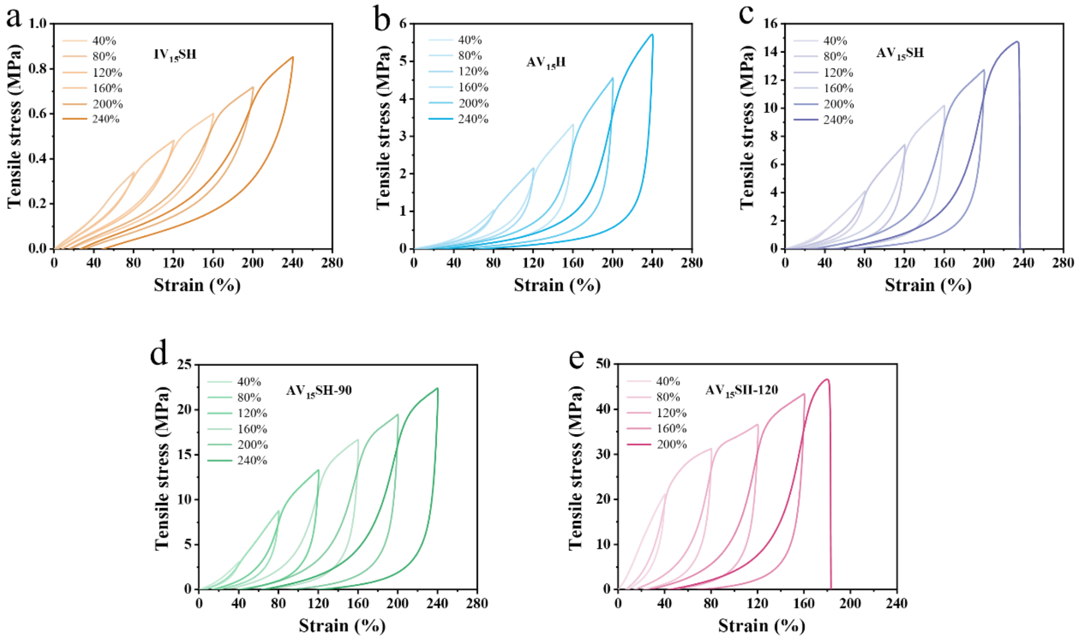

Figure S21. Successive tensile loading–unloading curves of a) $IV_{15}SH$, b) $AV_{15}H$, c) $AV_{15}SH$, d) $AV_{15}SH$-90 and e) $AV_{15}SH$-120 under different strains of 40–240 %.

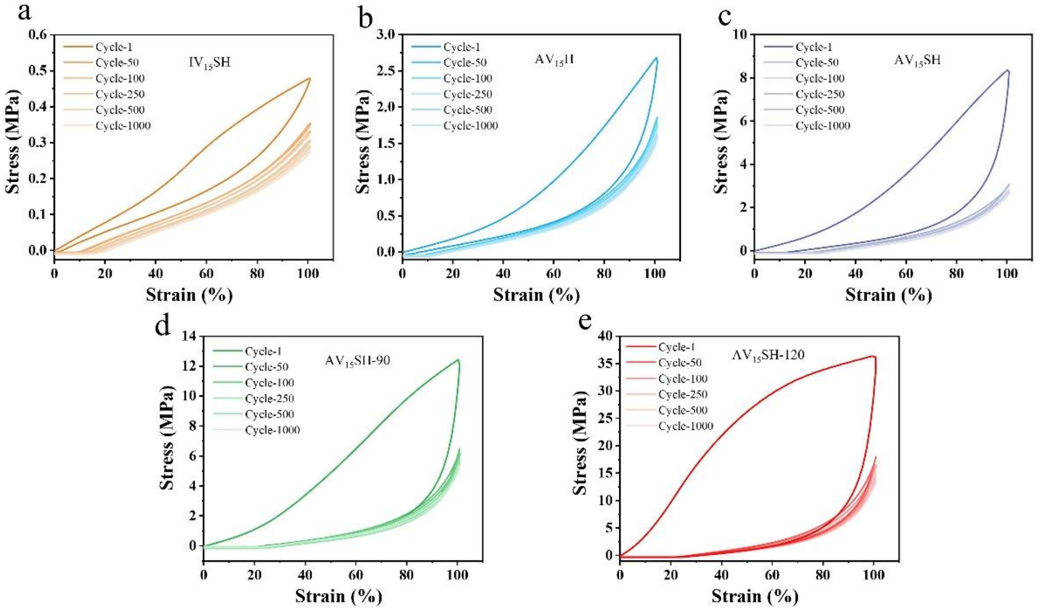

Figure S22. Cyclic stretching loading–unloading curves of a) $IV_{15}SH$, b) $AV_{15}H$, c) $AV_{15}SH$, d) $AV_{15}SH$-90 and e) $AV_{15}SH$-120 for over 1000 cycles at a predeterminate strain of 100 %.

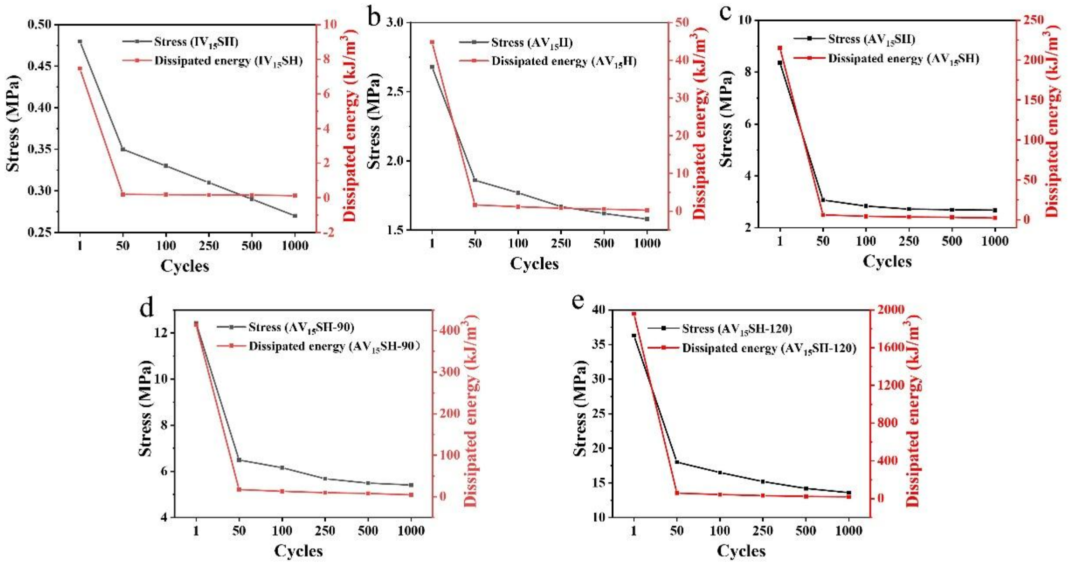

Figure S23. Stress and dissipated energy during 1000 cyclic loads of a) $IV_{15}SH$, b) $AV_{15}H$, c) $AV_{15}SH$, d) $AV_{15}SH$-90 and e) $AV_{15}SH$-120.

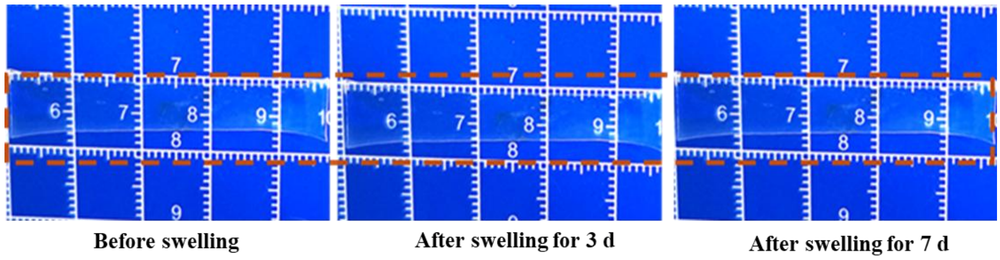

Figure S24. Photographs of $AV_{15}SH$-120 before and after swelling in H_2O.

Supplementary Tables

Sample	Crystal dimension (D, nm)	Inter-crystal spacing (L_1 V⊥, nm)	Inter-crystal spacing (L_2 H‖, nm)	Inter-laminar spacing (L_3, nm)	Inter-molecular spacing (L_4, nm)	Orientation degree
AV_{15}	4.48	9.39	10.06	0.4	0.7	0.82
AV_{15}-120	6.2	8.36	11.43	0.4	0.7	0.86
AV_{15}S-120	7.8	6.30	10.30	0.4	0.7	0.90

Table S1. Summary of crystal dimension (D), inter-crystal spacing (L_1), inter-crystal spacing (L_2), inter-laminar spacing (L_3), inter-molecular spacing (L_4) and Orientation degree.

Sample	Crystal dimension (D, nm)	Inter-crystal spacing (L_1 V⊥, nm)	Inter-crystal spacing (L_2 H‖, nm)	Inter-laminar spacing (L_3, nm)	Inter-molecular spacing (L_4, nm)	Crystallinity (%)	Orientation degree
IV_{15}SH	5.9	18.4	17.4	0.42	-	12.63	0
AV_{15}H	6.1	16.1	15.2	0.43	0.7	14	0.5
AV_{15}SH-120	7.6	8.5	10.3	0.46	0.7	34.3	0.87

Table S2. Summary of crystal dimension (D), inter-crystal spacing (L_1), inter-crystal spacing (L_2), inter-laminar spacing (L_3), inter-molecular spacing (L_4), crystallinity and orientation degree within the PVA hydrogel samples.
